# Reply: From Calcium Volume Correlations to Whole-Plaque Phenotyping: A Road Still to Be Traveled

**DOI:** 10.1016/j.jacadv.2026.103425

**Published:** 2026-11-02

**Authors:** Ruben G.A. van der Waerden, Niels van Royen, Rick H.J.A. Volleberg

**Affiliations:** a Department of Cardiology, Radboud University Medical Center, Nijmegen, the Netherlands; b Diagnostic Image Analysis Group, Radboud University Medical Center, Nijmegen, the Netherlands

We thank Dr Papazoglou and colleagues for their thoughtful engagement with our work^1^ and for situating our findings within the broader context of the coronary calcium paradox.

The correspondents question whether our findings, which focused primarily on calcium burden, capture the full complexity of calcific plaque. So far, calcium burden has been the principal metric used to characterize coronary calcification, in part because it can be readily quantified with clinically available imaging modalities, including optical coherence tomography (OCT) as used in our study. OCT allows quantification of calcium arc, depth and thickness (to a certain extent), and more recently also volume and burden through automated image analysis.^2^ We acknowledge, however, that the relatively small penetration depth limits visualization of the abluminal plaque content in very thick calcific lesions. We appreciate the suggestion of combining intravascular ultrasound with OCT and acknowledge the potential of multimodality imaging,^3^ although it should be noted that intravascular ultrasound is also limited in quantifying calcium burden and other deep plaque characteristics in these instances, as ultrasound waves are unable to penetrate calcium.

The correspondents raise an important point: calcium burden alone may not capture the full picture. In our view, this is where the true value of multimodality imaging lies—being able to evaluate distinct and complementary aspects of calcific plaques, including its activity, composition, morphology, and related biomechanics, as also alluded to by the correspondents. For instance, ^18^F-sodium fluoride-positron emission tomography can provide insights into calcification activity, whereas finite element analysis applied to intravascular imaging data can characterize the biomechanical stress associated with calcification.^4^, ^5^ The correspondents' unpublished ex vivo micro–computed tomography and Raman spectroscopy data could provide a valuable additional dimension by addressing calcium composition. We note, however, that this compositional evidence currently relies on histology-defined plaque instability in ex vivo specimens.

Ultimately, the key question is whether the integration of these differential aspects can improve lesion-level risk stratification.

Moving forward, we welcome the suggestion to investigate the spatial relationship between calcium deposits and adjacent plaque components. Because manual spatial assessment of this kind is highly labor-intensive, we believe our artificial intelligence–based segmentation model^2^ could reliably support such analysis in future work.

Overall, we agree the field should move toward a more comprehensive assessment of calcium phenotype that integrates its activity, biomechanics, composition and morphology—an endeavor that will likely require multimodality imaging. Step-by-step, these advances will take us further down the road toward a more complete understanding of the interplay between calcium and plaque vulnerability.
